# Adverse childhood experience and persistent insomnia during emerging adulthood: do positive childhood experiences matter?

**DOI:** 10.1186/s12889-024-17774-w

**Published:** 2024-01-24

**Authors:** Meng-Hsuan Wu, Chi Chiao, Wen-Hsu Lin

**Affiliations:** 1 Institute of Health and Welfare Policy, College of Medicine, National Yang Ming Chiao Tung University, No. 155, Sec. 2, Linong St., Beitou Dist, 112304 Taipei City, Taiwan; 2 Institute of Public Health, College of Medicine, National Yang Ming Chiao Tung University, No. 155, Sec. 2, Linong St., Beitou Dist, 112304 Taipei City, Taiwan

**Keywords:** ACE, PCE, Persistent insomnia symptoms, Resilience theory, Compensatory model, Protective model, Challenge model, Early Adolescence, Emerging Adulthood, Longitudinal study, Taiwan Youth Project

## Abstract

**Background:**

Adverse childhood experiences (ACE) have been documented to have long-term impacts on sleep disturbances. However, less is known about how ACE co-occurs with positive childhood experiences (PCE) and modulate their effects on adult sleep disturbances, particularly in the context of persistent insomnia. Building on resilience theory, this study aims to examine the interplay between ACE and PCE and their effects on persistent insomnia during emerging adulthood.

**Methods:**

A total of 2,841 emerging adults were recruited from the Taiwan Youth Project. Persistent insomnia during emerging adulthood was assessed using two adult surveys (mean age = 19.8 and 21.9). The ACE (10 items) and PCE (7 items) were obtained from the baseline survey (mean age = 13.8). A series of logistic regression analyses were conducted.

**Results:**

Among the emerging adults, 29.22% had persistent insomnia. Consistent with the compensatory model, ACE and PCE exerted opposing effects on persistent insomnia during emerging adulthood. In line with the protective model, the negative effect of ACE is mitigated when individuals have high PCE. However, consistent with the challenge model, the protective effect of PCE on persistent insomnia was inhibited in individuals with four or more ACE.

**Conclusions:**

PCE serves as a protective factor, shielding emerging adults from the adverse effects of ACE on persistent insomnia. It is essential to prioritize positive experiences during early life to promote lifelong sleep health.

**Supplementary Information:**

The online version contains supplementary material available at 10.1186/s12889-024-17774-w.

## Background

Many adults worldwide suffer from insomnia [1, 2], which significantly affects their health [3, 4], quality of life [5], and productivity, posing a direct risk to their income and further burdening national economic systems [6, 7]. Insomnia is often considered a situational problem triggered by life events, with remission occurring once the triggering event is resolved [8]. However, some individuals develop persistent insomnia due to recurrent sleep disturbances associated with stressful events [8]. Therefore, it is imperative that these individuals receive increased attention, coupled with a thorough examination of the early contributing factors and subsequent development of effective interventions.

Adverse childhood experiences (ACE) have been shown to have traumatic and lasting effects on health and behavior across the lifespan [9, 10]. An increased likelihood of poor sleep quality, sleep disruption, and psychiatric sleep disorders in adulthood has been found to be associated with ACE [11–14]. Early stressful life events in ACE research were initially focused only on the family context (i.e., abuse, neglect, and family dysfunction) [15] and have recently been expanded to encompass school and community contexts (i.e., peer victimization, peer isolation, and community violence) [16]. Although numerous studies have found a link between ACE and sleep disturbances [11–14], few have investigated persistent insomnia. According to the theory of trauma-induced chronic insomnia [17, 18], traumatic events trigger hyperarousal, resulting in immediate insomnia symptoms. Even after these events subside, some individuals, particularly those exposed to severe trauma, may develop a fear of sleep, fear of re-experiencing traumatic events through nightmares, or lose control due to dysfunctional sleep beliefs. These factors increase arousal and emotional distress, ultimately leading to severe chronic insomnia.

Although the comprehensive mechanisms of trauma-induced chronic insomnia have been extensively studied, coping factors in this condition have remained less explored. Based on the concept of resilience theory [19, 20], it is essential to recognize that adverse and positive events regularly coexist in daily life, contributing to an individual’s resilience and ability to adapt to life’s processes and challenges. Three models have been developed using this theory [20]. First, the compensatory model emphasizes the direct and beneficial effects of positive experiences on outcomes, which serve as a counterbalance to compensate for risks. Second, the protective model suggests that positive experiences can act as a buffer against the negative effects of adversity on outcomes. Third, the challenge model suggests that the beneficial effects of positive experiences may diminish when individuals experience excessive adversity.

An increasing body of research has explored the interplay between positive childhood experiences (PCE) and ACE and their influence on later health [21–25]. The term PCE was initially coined and collected by Bethell et al. [21], focusing on crucial factors for child development, particularly interactions and attachments between children and their family members (e.g., feeling supported by family during difficult times), schools (e.g., experiencing a sense of belonging at school), and communities (e.g., enjoying participating in community activities). These PCE represent a potential reservoir of resilience that children develop and possess [21]. In the field of sleep health, a cross-sectional study based on resiliency theory found a direct and reverse association between adult sleep difficulties and recalled ACE and PCE, consistent with the compensatory model [22]. Furthermore, the adverse effect of ACE on adult sleep problems was mitigated when PCE was high, consistent with the protective model [22]. However, the protective effect of PCE was diminished when an individual experienced four or more ACE, echoing the challenge model [22]. Another study conducted in a Chinese cohort, which shares a similar sociocultural context with Taiwan, provided further clarification regarding the longitudinal protective effects of PCE on insomnia in adults with ACE, even after accounting for factors such as post-traumatic stress disorder (PTSD) and depressive symptoms [23]. Nonetheless, the influence of the interplay between PCE and ACE on persistent insomnia remains unclear.

Building on the framework of resilience models [20], this study used a cohort study to investigate the longitudinal relationships between ACE, PCE, and persistent insomnia during emerging adulthood. Emerging adulthood (EA), defined as a transitional developmental phase bridging the gap between adolescence and adulthood, is characterized by significant and challenging life transitions [26]. With these emerging changes and the continuity of development and maturing, some argue that EA is the second most important life stage after infancy [27]. Sleep problems (e.g., insomnia) may be particularly prevalent during EA, as individuals need to learn how to manage life independently, often involving leaving home for college [28]. According to previous studies, sleep patterns tend to be disrupted when adopting a new lifestyle [29, 30], which may lead to sleep problems. In contrast, children and adolescents often stay at home, resulting in a relatively stable lifestyle. Moreover, a recent review [31] demonstrated that the majority of previous studies focusing on the effect of early adversity (e.g., maltreatment) on later-life sleep problems used samples of children and adolescents. Therefore, the current understanding of the association between ACE and sleep problems, such as insomnia, needs improvement. In view of this, understanding the interaction between ACE and PCE to influence insomnia in emerging adulthood is of paramount importance. This study hypothesized that ACE and PCE have direct and reverse effects on persistent insomnia during emerging adulthood, consistent with the compensatory model (H1). Furthermore, it was hypothesized that individuals with higher PCE possess the ability to mitigate the negative effects of ACE on later persistent insomnia, supporting the protective model (H2). Conversely, higher ACE levels were expected to hinder the protective effect of PCE on later persistent insomnia, consistent with the challenge model (H3).

## Methods

### Data and sample

This study used data from the Taiwan Youth Project (TYP), a panel dataset created by the Institute of Sociology, Academic Sinica, Taiwan. Two cohorts of adolescents from northern Taiwan, grade 7 (mean age = 13) and grade 9 (mean age = 15), were recruited using a hierarchical multistage random cluster sampling design. After the initial survey in 2000, participants were followed up annually until 2017. Detailed information on TYP sampling is provided elsewhere [32]. This study received permission to use the TYP datasets for research purposes from Academic Sinica in Taiwan and was also approved by the Research Ethics Committee of National Yang Ming Chiao Tung University (Taipei, Taiwan) (YM108160EF). The TYP datasets are publicly available on the Survey Research Data Archive.

**Fig. 1:**
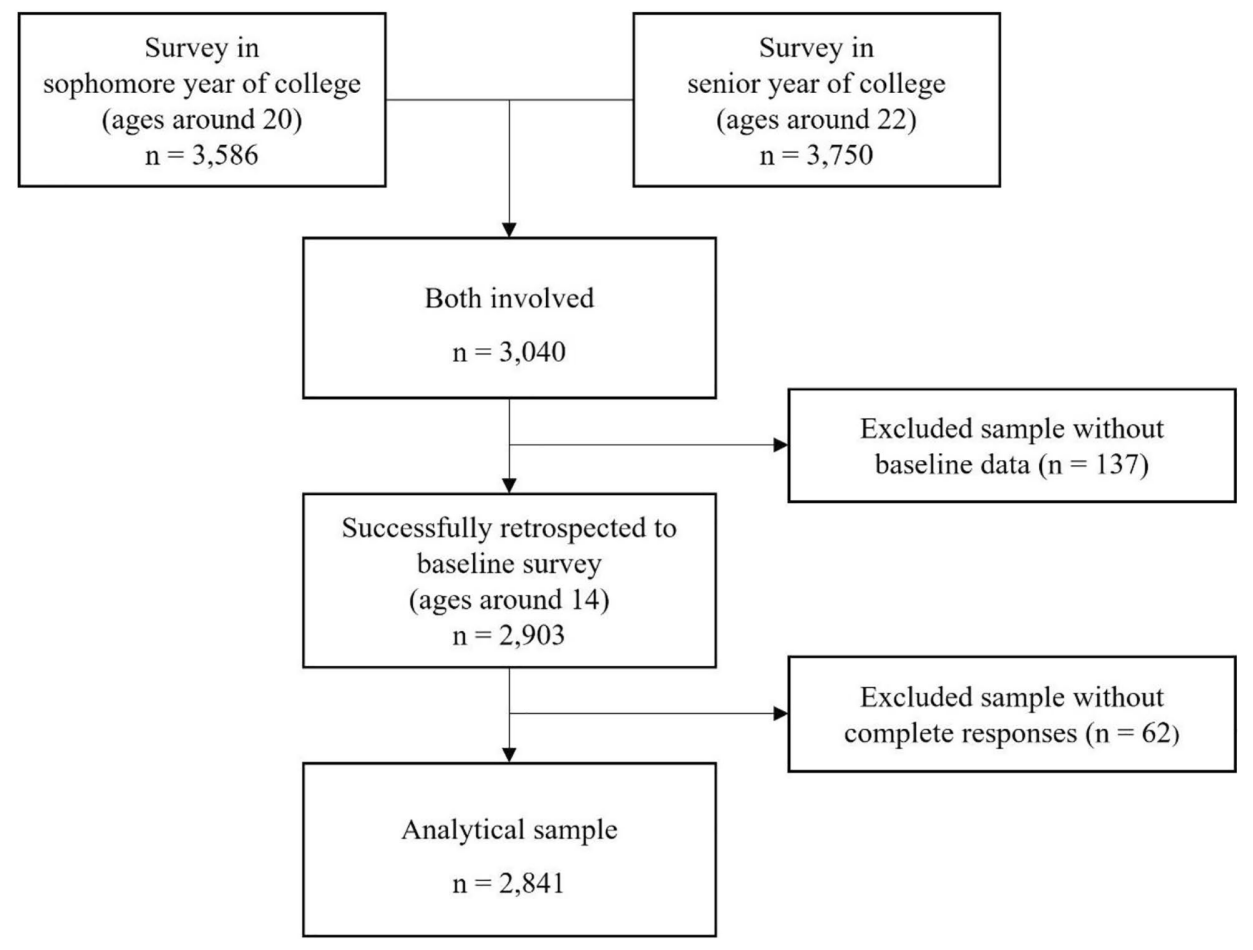
Sample flow, *n* = 2,841

The current study used TYP datasets retrospectively through three steps. First, we employed surveys when participants were in their sophomore and senior years of college (i.e., 20 and 22 years old), resulting in the inclusion of 3,040 individuals. Second, these participants were then matched to their ACE and PCE data from the baseline surveys, and 2,903 participants were successfully matched. Finally, participants who did not respond to ACE, PCE, and persistent insomnia (missing rate < 1%) were also excluded. This process ultimately yielded 2,841 emerging adults with complete responses for analysis (See Fig. 1 for detail sample flow). Although some participants were not included due to their lack of involvement in the baseline survey, these individuals did not differ from the analytical sample in terms of persistent insomnia. However, those with non-response to important variables were more likely to be male and younger than the analytic sample. In order to undergird our main analyses in this study, we also conducted a sensitivity analysis by imputing missing values using mean replacement (see Table S4 in online supplement).

### Measures

#### Persistent insomnia during emerging adulthood

Persistent insomnia was assessed using surveys administered at ages 20 and 22. In these two surveys, participants were asked about their difficulty falling asleep in the past week, and the severity was rated on a five-point scale. The original responses were simplified to the binary values of 0 (never) and 1 (mild to extremely severe). The study then cross-tabulated the recoded measures from both surveys to produce the final binary outcome. Participants who reported experiencing insomnia at both time points were grouped together and coded as 1, whereas all other participants were coded as 0.

According to the literature, emerging adulthood spans from 18 to 25 [26]. Our measure is deemed reasonable for two primary reasons. Empirically, only these two waves of the survey utilized a complete and identical measure of insomnia during emerging adulthood in TYP. Second, in Taiwan and many neighboring countries (e.g., South Korea), adolescents at the 12th grade (around 18) face tremendous academic-related stress due to entrance examinations [33]. Consequently, including this idiosyncratic time in our analysis may risk distorting the regular insomnia pattern during emerging adulthood.

#### Adverse childhood experiences (ACE)

The ACE, based on the revised CDC-Kaiser ACE study [16], was assessed using baseline data (mean age, 14 years). Owing to data limitations, this study used 10 items to module the ACE: emotional abuse, physical abuse, emotional neglect, parental divorce/separation, household substance abuse, household mental illness, household incarcerated members, peer isolation, community safety, and household poverty. Both participant and parent responses were used to provide a comprehensive understanding of the participants’ experiences. For example, data on household dysfunction was collected from parent baseline surveys. Each ACE item was transformed into a dummy variable: 1 indicates that an individual experienced an adverse event, and 0 indicates the absence of that experience. When items were measured on a scale, a cutoff for extreme values (e.g., severe/very severe) was applied to identify severe adversity (see Table S1 in online supplement). The sum of these 10 dichotomous items produced an ACE score ranging from 0 to 10.

#### Positive childhood experiences (PCE)

PCE was assessed in a baseline survey using seven advantageous items [21]. These items included (1) being able to talk about feelings with your family, (2) having family support during difficult times, (3) feeling safe and protected by an adult in your family, (4) having a sense of belonging at high school, (5) being supported by friends, (6) having at least two non-parental adults who take a genuine interest in you, and (7) enjoying participating in community traditions. Participants were asked to report how often they experienced each statement or how well it described them. Similar to the coding approach for the ACE, each individual PCE item was dichotomized, and the sum of these seven items was used to measure PCE, with a higher score indicating more positive experiences (see Table S2 in online supplement).

#### Covariates

Demographics, including age, gender, parental education, and growing-up location, were adjusted for. Additionally, this study controlled for substance use, self-esteem, depressive symptoms, and insomnia symptoms during adolescence, all of which are important predictors of adult insomnia [34, 35] (see Table S3 in online supplement).

### Statistical analyses

This study employed a series of logistic regression analyses to test three hypotheses based on resilience theory models [20]. For the compensatory model (H1), accumulation of PCE and ACE were simultaneously tested in a regression analysis to determine whether they have independent and opposing effects on persistent insomnia. For the protective model (H2), the study aimed to examine whether the negative effect of ACE on persistent insomnia was diminished in those with higher PCE scores. To achieve this, the sample was further stratified into two groups using a mean split of PCE scores (> 3 vs. ≤3), as established in a previous study [22]. Finally, for the challenge model (H3), the sample was further divided based on a cutoff ACE score of four (ACE ≥ 4 vs. ACE < 4) [15] to investigate whether the protective effect of PCE was hindered among those with higher ACE scores. All regression models were adjusted for covariates (variance inflations (VIF) in all logistic regressions were < 2.5 [36]). As there was no clear cutoff for PCE, a sensitivity analysis was conducted using three different cutoffs to explore the conditions associated with the highest PCE: 1SD (PCE ≥ 4), top 20% (PCE ≥ 5), and the highest two groups (PCE ≥ 6) [21]. All regression analyses were performed with STATA version 16.0.

## Results

### Sample characteristics

Table 1 lists the sample characteristics. Gender was almost evenly distributed (50.79% women), and the mean age was 21.82 years (standard deviation (SD) = 0.68). During emerging adulthood, 29.22% of participants reported persistent insomnia. Participants had a mean ACE score of 1.26 (SD = 1.36), with 64.24% of the participants experiencing at least one ACE and 7.29% experiencing four or more adversities. The average PCE score was 3.08 (SD = 1.60), and 7.78% of participants had six or more PCE. Among these emerging adults, approximately one-third (37.38%) resided in urban areas (Taipei City), and 13.94% had parents with high educational attainment (i.e., college or higher). Additionally, 8.52% reported involvement in substance use (e.g., cigarettes or alcohol). On average, self-esteem was rated at 2.74 on a scale from 1 (lowest) to 4 (highest), while the average level of depressive symptoms was 0.60 on a scale from 0 (none) to 3.88 (most severe). Approximately 38.79% of participants reported experiencing insomnia symptoms during adolescence.

**Table 1:** Sample characteristics, *n* = 2,841

Variables	Percent/mean(SD)
Age (range 21–26)	21.82 (0.68)
Female (%)	50.79
**Emerging adulthood (aged around 20 to 22)**	
Persistent insomnia (%)	29.22
**Early adolescence (aged around 14)**	
Adverse childhood experiences (range 0–8)	1.26 (1.36)
Positive childhood experiences (range 0–7)	3.08 (1.60)
Location (%)	
Taipei city	37.38
New Taipei city	37.73
I-Lan county	24.89
Highest parental education (college or above) (%)	13.94
Ever substance use (%)	8.52
Self-esteem (range 1–4)	2.74 (0.56)
Depressive symptoms (range 0-3.88)	0.60 (0.59)
Ever insomnia symptom (%)	38.79

Note: SD = Standard Deviation

### Childhood experiences and persistent insomnia during emerging adulthood

Table 2 displays the results of testing the compensatory model of resilience theory. Individuals with higher ACE scores were more likely to experience persistent insomnia during emerging adulthood (adjusted odds ratio (AOR) = 1.12, 95% CI: 1.05–1.19). Additionally, regardless of ACE scores, PCE also exhibited a significant effect on persistent insomnia as an independent protective factor (AOR = 0.93, 95% CI: 0.88–0.98).

**Table 2:** The compensatory model of resiliency theory ^a^: PCE, ACE and persistent insomnia during emerging adulthood ^b^, *n* = 2,841

Variables	Persistent insomnia during emerging adulthood (vs. no)
	AOR [95% CI]
Age	1.07 [0.94, 1.21]
Female (vs. male)	1.26 [1.06, 1.50]**
**Early adolescence** ^**c**^	
Location (vs. Taipei city)	
New Taipei city	1.05 [0.86, 1.23]
I-Lan County	0.89 [0.71, 1.12]
Parental education (vs. below college)	0.73 [0.57, 0.95]*
Substance use (vs. never)	0.75 [0.55, 1.02]+
Self-esteem	1.04 [0.88, 1.22]
Depressive symptoms	1.45 [1.24, 1.70]**
Insomnia symptom	1.77 [1.48, 2.11]**
ACE	1.12 [1.05, 1.19]**
PCE	0.93 [0.88, 0.98]*

Note: *p* < 0.1 (+); *p* < 0.05 (*); *p* < 0.01 (**)

AOR = Adjusted Odds Ratio; ACE = Adverse Childhood Experience; PCE = Positive Childhood Experience

^a^ Compensatory model of resilience theory: PCE and ACE had independently reverse effect on later persistent insomnia, with PCE having a protective effect and ACE having a negative effect

^b^ Persistent insomnia was measured when subjects were around 20 and 22 years old

^c^ Early adolescence in this study was defined as the period when the subjects were around 14 years old

### PCE mitigates the effect of ACE on persistent insomnia during emerging adulthood

Table 3 presents the results of testing the protective resilience theory model. Overall, higher PCE appeared to mitigate the negative effects of ACE on persistent insomnia during emerging adulthood. Specifically, among those with a high PCE (i.e., > 3), ACE showed no significant long-term relationship with persistent insomnia. Conversely, in the low-PCE group (i.e., ≤ 3), ACE was significantly associated with persistent insomnia (AOR = 1.15, 95% CI: 1.06–1.24).

**Table 3:** The protective model of resiliency theory ^a^: PCE, ACE and persistent insomnia during emerging adulthood ^b^, *n* = 2,841:

Variables	Stratification of PCE scores by mean-split		
	PCE ≤ 3 *n* = 1,754		PCE > 3 *n* = 1,087
	Persistent insomnia during emerging adulthood (vs. no)		Persistent insomnia during emerging adulthood (vs. no)
	AOR [95% CI]		AOR [95% CI]
Age	1.05 [0.90, 1.22]		1.10 [0.89, 1.36]
Female (vs. male)	1.28 [1.03, 1.58]*		1.20 [0.90, 1.60]
**Early adolescence** ^**c**^			
Location (vs. Taipei city)			
New Taipei city	1.11 [0.86, 1.42]		0.96 [0.69, 1.32]
I-Lan County	1.00 [0.76, 1.32]		0.73 [0.49, 1.09]
Parental education (vs. below college)	0.69 [0.49, 0.98]*		0.75 [0.50, 1.11]
Substance use (vs. never)	0.81 [0.57, 1.15]		0.60 [0.30, 1.21]
Self-esteem	1.10 [0.89, 1.36]		0.89 [0.69, 1.15]
Depressive symptoms	1.42 [1.16, 1.74]**		1.47 [1.14, 1.91]**
Insomnia symptom	1.59 [1.27, 1.99]**		2.13 [1.58, 2.87]**
ACE	1.15 [1.06, 1.24]**		1.07 [0.96, 1.20]

Note: *p* < 0.1 (+); *p* < 0.05 (*); *p* < 0.01 (**)

AOR = Adjusted Odds Ratio; ACE = Adverse Childhood Experience; PCE = Positive Childhood Experience

^a^ Protective model of resilience theory: higher PCE mitigated the negative effects of ACE on later persistent insomnia

^b^ Persistent insomnia was measured when subjects were around 20 and 22 years old

^c^ Early adolescence in this study was defined as the period when the subjects were around 14 years old

### Higher ACE inhibited the effect of PCE on persistent insomnia during emerging adulthood

Table 4 illustrates the results of testing the resilience theory challenge model. Generally, higher ACE scores appeared to diminish the protective effect of PCE in emerging adulthood persistent insomnia. Specifically, among patients taking four or more ACE, PCE did not exhibit a significant protective effect against persistent insomnia. Conversely, individuals with relatively low levels of ACE (i.e., < 4) experienced a significant reduction in the risk of persistent insomnia when higher PCE was present (AOR = 0.93, 95% CI: 0.88–0.99).

**Table 4:** The challenge model of resiliency theory ^a^: PCE, ACE and persistent insomnia during emerging adulthood ^b^, *n* = 2,841:

Variables	Stratification of ACE scores by four or more		
	ACE < 4 *n* = 2,634		ACE ≥ 4 *n* = 207
	Persistent insomnia during emerging adulthood (vs. no)		Persistent insomnia during emerging adulthood (vs. no)
	AOR [95% CI]		AOR [95% CI]
Age	1.08 [0.94, 1.22]		1.00 [0.65, 1.54]
Female (vs. male)	1.23 [1.02, 1.47]*		1.57 [0.87, 2.86]
**Early Adolescence**			
Location (vs. Taipei city)			
New Taipei city	1.07 [0.87, 1.31]		0.88 [0.45, 1.71]
I-Lan County	0.89 [0.70, 1.13]		0.80 [0.35, 1.83]
Parental education (vs. below college)	0.69 [0.52, 0.91]**		1.29 [0.48, 3.47]
Substance use (vs. never)	0.71 [0.50, 1.01]+		1.13 [0.52, 2.45]
Self-esteem	1.04 [0.87, 1.25]		0.95 [0.59, 1.55]
Depressive symptoms	1.59 [1.34, 1.89]**		1.09 [0.75, 1.59]
Insomnia symptom	1.73 [1.44, 2.09]**		1.76 [0.96, 3.23]+
PCE	0.93 [0.88, 0.99]*		0.82 [0.66, 1.02]+

Note: *p* < 0.1 (+); *p* < 0.05 (*); *p* < 0.01 (**)

AOR = Adjusted Odds Ratio; ACE = Adverse Childhood Experience; PCE = Positive Childhood Experience

^a^ Challenge model of resilience theory: higher ACE diminished the protective effects of PCE on later persistent insomnia

^b^ Persistent insomnia was measured when subjects were around 20 and 22 years old

^c^ Early adolescence in this study was defined as the period when the subjects were around 14 years old

### Sensitivity analysis

We conducted two sensitivity analyses to further strengthen our main results. First, as mentioned, we discarded some of the samples because of non-responses. We conducted all the analyses with imputed values for these samples. The results were very similar, with only minor changes in the coefficients (see Table S4 in the online supplement). For example, ACE (AOR = 1.11, 95% CI: 1.04–1.18) and PCE (AOR = 0.93, 95% CI: 0.88–0.99) were still significantly associated with persistent insomnia. Second, we employed different cutoffs for PCE. In this part of the sensitivity analyses, we used three additional cutoffs: 1 SD above the mean (PCE = 4 or higher) vs. others; top 20% (PCE = 5 or higher) vs. others; and top two categories (PCE = 6 or higher) vs. others. When stratifying the sample using these cutoffs, the protective effect of PCE remained consistent with that in the main analyses (see Table S5 in online supplement). For example, in the high-PCE group (PCE ≥ 6), the negative effects of ACE on persistent insomnia during emerging adulthood were not statistically significant. Conversely, in the low-PCE group, ACE remained a risk factor for more persistent insomnia (AOR = 1.11, 95% CI: 1.04–1.19).

## Discussion

Using a cohort sample from the TYP, this study found that the interplay between ACE and PCE and persistent insomnia during emerging adulthood is consistent with the principles of resilience theory [19, 20]. These findings support our first hypothesis that ACE and PCE have direct and inverse effects on persistent insomnia in emerging adulthood. Specifically, PCE maintained its protective effect regardless of the number of ACE experienced, consistent with the compensatory model. Furthermore, our second hypothesis is supported, as PCE serves as a buffer that mitigates the negative impact of ACE on later persistent insomnia, consistent with the protective model. Moreover, our third hypothesis was confirmed, illustrating that the protective influence of PCE on later persistent insomnia was impeded when ACE experiences were excessive, consistent with the challenge model.

Expanding on prior studies of ACE and adult sleep problems [11–14], our findings support the notion that ACE has a lasting negative impact on sleep, particularly contributing to persistent insomnia in emerging adults. Consistent with the theory of trauma-induced chronic insomnia [17, 18], a state of sustained hyperarousal serves as the primary mechanism by which adults who have experienced severe early adversity develop a fear of sleep that subsequently leads to persistent insomnia. Furthermore, early social stress impairs adolescent brain development and neuroendocrine responses [37]. Individuals who experience these adversities may have elevated levels of stress hormones (e.g., cortisol), which can disrupt sleep [37]. Although our results supported the cumulative risk approach commonly found in ACE literature (i.e., adversity happening in tandem), some previous studies found that there might be a nuanced influence of ACE on later health [38, 39]. We, therefore, conducted further analyses to see if such is the case in understanding emerging adults’ persistent insomnia (Table S6 in online supplement). The results showed a similar pattern: experiencing adversity early in life is detrimental to later health. For example, we found that family dysfunction (AOR = 1.31, *p* < 0.01) and new ACE items (AOR = 1.25, *p* < 0.05) [16] were significantly related to a higher risk of persistent insomnia during emerging adulthood but not maltreatment.

In our study, however, PCE was found to serve as a protective factor against persistent insomnia, regardless of early adversity. This supports the compensatory model of resilience theory [19, 20] and is consistent with a previous study [21] that found PCE to be positively associated with improved health through social support. Moreover, our study demonstrates that PCE has a potential moderating effect on ACE and sleep. Based on the stress paradigm [40, 41], social support acts as a buffer against the negative health effects of major life events and chronic stressors, and this buffering effect, in turn, persists across life stages as a kind of “social fund” [28, 42]. The net protection or buffering role in the stress paradigm highlights the significance of PCE in adolescent social contexts, leading to the development of resilience. Resilience enables individuals to mobilize resources and helps them adapt well when facing co-occurring adversities [19]. Therefore, in addition to its direct protective effect, we estimated that PCE has a nonlinear association with sleep hygiene in emerging adults.

Nevertheless, in the context of persistent insomnia during emerging adulthood, the protective effect of PCE appears to be diminished when individuals face extreme early adversity (i.e., ≥ 4 ACE), which is consistent with the challenge model of resilience theory [19, 20] and previous studies in other health domains [22]. The challenge model underscores the benefits of modest risks in learning to overcome challenges when accompanied by adequate resources and support; however, overwhelming risks are detrimental to coping development [19, 20]. Despite this, based on the Health Outcomes from Positive Experiences (HOPE) framework designed to develop preventive strategies, particularly in adverse contexts, to promote attachment and resilience in child health [43], early studies suggested that children with high ACE still have the opportunity to achieve positive outcomes when they report feeling supported and having someone to confide in within their families during challenging times [44]. In addition to remediation, early home visitation, recommended by the US Advisory Board on Child Abuse and Neglect, has been proven as a promising approach to reduce or prevent early adversity [15]. Specialists can assist new parents in fostering healthy interactions with their children, reducing the risk of ACE development and providing long-term psychosocial benefits for both childhood and parenthood [15]. Overall, prioritizing the improvement of parent-child communication is essential.

The HOPE framework provides systematic recommendations for intervention policies, including (1) secure and stable environment, (2) nurturing relationship, (3) active social involvement, and (4) social and emotional intelligence [43]. The majority of these elements are comprehensive within PCE, with the exception of the last element. A secure and stable environment is crucial for the lifelong promotion of healthy sleep. This is closely followed by the quality of relationships, whether between children and their parents [28] or peers [45], and community social engagement [46]. All of these factors have been found to be positively associated with sleep hygiene, both in the short and long terms. Furthermore, additional analysis in this study revealed that children learning in a positive school environment were less likely to experience persistent insomnia during emerging adulthood. This environment was characterized by genuine interest from at least two non-parent adults, peer support, and a sense of school belonging. These findings persisted even when exposed to early adversities (See Table S7 in online supplement).

This study has several strengths. First, to the best of our knowledge, this study is the first to extend resilience theory to examine the interplay between ACE and PCE in persistent insomnia during emerging adulthood. Our measure of persistent insomnia can be viewed as a pre-symptomatic stage of chronic insomnia, which is classified as a mental disorder in the Diagnostic and Statistical Manual of Mental Disorders– Fifth Edition (DSM-V) [47]. Therefore, findings regarding the effects of ACE and PCE on persistent insomnia have implications for early detection and prevention. Second, unlike most ACE studies that use cross-sectional designs, this study utilized panel data, enabling the examination of longitudinal associations and the collection of ACE and PCE data when participants were under 18 years, thereby reducing recall bias. Moreover, the inclusion of parental data in this panel dataset provides a valuable opportunity to obtain a more realistic assessment of ACE, particularly with respect to information related to family dysfunction (i.e., parental divorce or separation, household mental illness, household substance use, and incarcerated household members).

### Limitations

This study still has several limitations. First, all variables in this study relied on self-reported data, potentially introducing common method bias (i.e., all data came from self-reports). While this may pose a threat to our results, several previous studies have shown similar relationships between our main exposure and outcomes [48]. Additionally, Harman’s one-factor test yielded acceptable results; specifically, the explained variance of the first factor was less than 50% [49]. Consequently, the threat of common method bias may be minimal but cannot be entirely ignored. Second, the measurement of persistent insomnia during emerging adulthood cannot accurately represent the entire period owing to data limitations, relying on assessments at only two time points. Relatedly, this also indicates that our results may not be applicable to the entire emerging adult population. Future studies are encouraged to utilize more cohort datasets and employ more advanced measurement techniques to assess persistent (chronic) insomnia and test our preliminary findings using trajectory models or clinical databases. Third, this study can only explain the main effects of longitudinal associations and cannot make causal inferences. Therefore, future longitudinal studies are strongly encouraged to explore potential mediators to elucidate the mechanisms underlying ACE, PCE, and persistent insomnia. Additionally, researchers should consider experimental or interventional approaches to elucidate causal relationships. Finally, the study used a sample from a Taiwanese cohort, raising concerns regarding its generalizability.

## Conclusions

Emerging adults with persistent insomnia have ACE and PCE. In addition to the opposing effects of ACE and PCE on persistent insomnia, PCE acts as a modifier that buffers the negative effects of ACE on insomnia. However, the buffering effect of PCE may be inhibited during challenging periods, with severe adverse effects. It is noteworthy that although it is worthwhile to investigate the development of prevention strategies for PCE, these strategies should be adapted under different scenarios, especially if individuals have ever suffered from miserable experiences. Efforts to reduce ACE and promote PCE will help individuals build more robust resilience and foster healthier sleep development.

### Electronic supplementary material

Below is the link to the electronic supplementary material.

Supplementary Material 1

## Data Availability

No datasets were generated or analysed during the current study.

## Abbreviations

ACE: Adverse Childhood Experiences
PCE: Positive Childhood Experiences
PTSD: Post-traumatic Stress Disorder
DSM-V: Diagnostic and Statistical Manual of Mental Disorders– Fifth Edition
TYP: Taiwan Youth Project
