# Supplementary material for: Adverse childhood experience and persistent insomnia during emerging adulthood: do positive childhood experiences matter?
Source: BMC Public Health. 2024 Jan 24;24:287. doi: 10.1186/s12889-024-17774-w (PMC10809570; doi:10.1186/s12889-024-17774-w)
Supplement: Supplementary file 1 — Supplementary Material 1 [file 12889_2024_17774_MOESM1_ESM.docx]

**Supplement**

| Table S1. ACE in the Taiwan Youth Project (TYP) baseline survey, referring to the revision of Kaiser ACE Study (Finkelhor et al., 2015). | | | | |
| --- | --- | --- | --- | --- |
| ACE | | Kaiser’s Definition | TYP W1 Question/answer | Coding (#step) |
| 1 | Emotional abuse | A parent or other adult in the household **often or very often** swore at me, insulted me, put me down, or humiliated me **OR** acted in a way that made me afraid that I might be physically hurt. | [Y] How often did your mom/dad … when you were with her/him in the last month?  Q1: shout and yell at you furiously  Q2: scream, bark, or scold you  Ans. 1.always 2.nearly always 3.often 4.half of the time 5.seldom 6.nearly never 7.never | #1. Q1&Q2 recode  1=always/nearly always  0=others  #2. Maximization  1=at least one  0=never experienced |
| 2 | Physical abuse | A parent or other adult in the household **often or very often** pushed, grabbed, slapped, or threw something at me **OR ever** hit me so hard that I had marks or were injured. | [Y] How often did your mom/dad … when you did something wrong in daily lives?  Q1: spank you  Q2: use a belt or something of the sort to whip you  Ans. 1.always 2.nearly always 3.half of the time 4.nearly never 5.never  Q3: [Y] how often did your mom/dad pinch, hit, or punch you when you were with her/him in the last month?  Ans. 1.always 2.nearly always 3.often 4.half of the time 5.seldom 6.nearly never 7.never | #1. Q1, Q2 & Q3 recode  1=always/nearly always  0=others  #2. Maximization  1=at least one  0=never experienced |
| 3 | Sexual assault | An adult or person at least 5 years older than me **ever** touched or fondled me or had me touch their body in a sexual way **OR** attempted or actually had oral, anal, or vaginal intercourse with me. | No corresponding questions | – |
| 4 | Emotional neglect | I **often or very often** felt that no one in my family loved ne or thought I were important or special **OR** my family didn’t look out for each other, felt close to each other, or supported each other. | Q1: [Y] how often did your mom/dad show that she/he really cared about you when you were with her/him in the last month?  Ans. 1.always 2.nearly always 3.often 4.half of the time 5.seldom 6.barely never 7.never  Q2: [Y] how often were your mom/dad concern about how you felt and any problems that were bothering you?  Ans. 1.often 2.sometimes 3.seldom 4.never | #1. Q1 & Q2 recode  Q1: 1=never/barely never  Q2: 1=never  #2. Maximization  1=at least one  0=never experienced |
| 5 | Physical neglect | I **often or very often** felt that I didn’t have enough to eat, had to wear dirty clothes, and had no one to protect me **OR** my parents were too drunk to take care of me or take me to the doctor if I needed it. | No corresponding questions | – |
| 6 | Parental divorce/ separation | A biological parent **ever** lost to me through divorce, abandonment, or other reason. | Q1: [P] what is your marital status now?  Ans. 1.first marriage 2.remarriage 3.divorced 4.widowed  Q2: [P] do you live with your spouse?  Ans. 1.yes 2.no  Q3: [Y] what is your parents’ marital status?  Ans. 1.live together 2.dad deceased 3.mom deceased 4.divorced 5.separeted 6.devorced but live together | #1. P ans. recode  1=Q1 [2/3/4] or Q2[2]  0=1^st^ marriage  Y ans. recode  1=others  0=parents live together  #2. Mainly use P ans.,  replace missing w/ Y ans. |
| 7 | Mother treated violently | My mother or stepmother was **often or very often** pushed, grabbed, slapped, or had something thrown at her **OR sometimes, often, or very often** kicked, bitten, hit with a fist, or hit with something hard **OR ever** repeatedly hit over at least a few minutes or threatened with a gun or knife. | No corresponding questions | – |
| 8 | Household substance abuse | I lived with anyone who was a problem drinker or alcoholic, or who used street drugs. | Q1: [P] have you noticed your spouse had a problem of binge-drinking or gambling since last year?  Ans. 1.yes 2.no  Q2: [P] have you had a problem of binge-drinking or gambling since last year?  Ans. 1.yes 2.no | #1. Q1&Q2 recode  1=yes  0=no  #2. Maximization  1=at least one  0=never experienced |
| 9 | Household mental illness | A household member was depressed or mentally ill, or a household member attempted suicide. | [P] Did you have the following symptoms last week? If yes, how severe is it?  Q1: headache. Q2: dizziness. Q3: stress gets you trouble getting your breath. Q4: temper outbursts that you could not control. Q5: loss the will to live. Q6: blame yourself for everything. Q7: soreness of your muscles. Q8: depression. Q9: loneliness. Q10: worry too much about things. Q11: feeling no interest in things. Q12: insomnia or sleeping worse. Q13: numbness/tingling pain in certain parts of the body. Q14: feeling weak in part of your body. Q15: feeling tense or keyed up. Q16: feeling laborious when doing anything. Q17: loss of sexual interest or pleasure.  Ans. 1.no 2.mild 3.moderate 4.severe 5.very severe | #1. Employing GSI  (Global Severity Index)  that averaging scores for all responded items  #2. Mean+1SD as a cut-off  (Dang et al., 2019)  1=had family mental ill  0= never experienced |
| 10 | Incarcerated household member | A household member went to prison. | Q1: [P] have you had a lawsuit or been arrested since last year?  Ans. 1.yes 2.no | #1. Recode  1=yes  0=no |
| 11 | Peer victimization | Other kids, including brothers or sisters, **often or very often** hit me, threatened me, picked on me or insulted me. | No corresponding questions | – |
| 12 | Peer isolation/  rejection | I **often or very often** felt lonely, rejected or that nobody liked me. | Q1: [Y] how many classmates are your good friends?  Ans. 1.almost everyone 2.most of the classmates 3. half of the classmates 4.a few of the classmates 5.nearly none  Q2: [Y] how often are you worried about being unpopular among your classmates?  Ans. 1.almost always 2.sometimes 3.seldom 4.almost never  Q3: [P] is your child unpopular or isolated by others?  Ans. 1.no 2.mild 3.moderate 4.serious | #1. Q1, Q2 & Q3 recode  Q1: 1=nearly none  Q2: 1=almost always  Q3: 1=serious/moderate  #2. Maximization  1=at least one  0=never experience |
| 13 | Expose to community violence | I lived for 2 or more years in a neighborhood that was dangerous, or where I saw people being assaulted. | Q1: [Y] do you feel the security around your residential area is good?  Ans. 1.very good 2.good 3.poor 4.very poor | #1. Recode  1=very poor/poor  0=others |
| 14 | Low socioeconomic status | There was a period of 2 or more years when my family was very poor or on public assistance. | Q1: [Y] have your family become poor since last year?  Ans. 1.yes 2.no | #1. Recode  1=yes  0=no |
| Note: ACE = Adverse Childhood Experience; Y = Youth version questionnaire; P = Parental version questionnaire | | | | |

| Table S2. PCE in the Taiwan Youth Project (TYP) baseline survey, referring to the study from Bethell and her colleagues (2019). | | | |
| --- | --- | --- | --- |
| PCE (Bethell’s definition) | | TYP W1 Question/answer | Coding (#step) |
| 1 | Able to talk to family about feelings | Q: [Y] how often did your mom/dad carefully listen to your opinions and viewpoints when you were with her/him in the last month?  Ans. 1.always 2.nearly always 3.often 4.half of the time 5.seldom 6.nearly never 7.never | #1. Recode  1=always/nearly always  0=others |
| 2 | Felt family stood by them during difficult times | Q: [Y] how much can you get comfort from your family when you experience setbacks in life?  Ans. 1.very much 2.somewhat 3.no really 4.not at all | #1. Recode  1=very much  0=others |
| 3 | Felt safe and protected by adult in difficult times | Q: [Y] did your mom/dad always there for you when you really need help?  Ans. 1.often 2.sometimes 3.seldom 4.never | #1. Recode  1=often  0=others |
| 4 | Had at least 2 non-parent adults who took genuine interest them. | Q: [Y] how many teachers have been willing to pay attention on you since you attended junior high?  Ans. 1.none 2.one 3.two 4.three 5.four 6.more than five | #1. Recode  1=two or more  0=none/one |
| 5 | Felt supported by friends | [Y] How much do you agree with the following description of your best friends?  Q1: they care about me.  Q2: they often help me when I am in need.  Q3: they always give me comfort when I experience setbacks.  Ans. 1.strongly agree 2.agree 3.disagree 4.strongly disagree | #1. Q1, Q2 & Q3 recode  1=strongly agree  0=others  #2. Maximization  1=at least one  0=never experienced |
| 6 | Felt a sense of belonging at high school | [Y] How much do you agree with the following statements regarding your school?  Q1: I like my school.  Q2: I don’t think I am a part of the school.  Q3: I am proud of my school.  Ans. 1.strongly agree 2.agree 3.disagree 4.strongly disagree | #1. Q1, Q2 & Q3 recode  1= strongly agree  0=others  #2. Maximization  1=at least one  0=never experienced |
| 7 | Enjoyed participating in community traditions | [Y] Have you participated in the following activities around your residence?  Q1: community sponsored activities.  Q2: religious activities (sponsored by community church or temple).  Q3: activities that make use of public facilities (e.g., read books in libraries, play basketball in the gym)  Ans. 1.yes 2.no | #1. Q1, Q2 & Q3 recode  1=yes  0=no  #2. Maximization  1=at least one  0=never experienced |
| Note: PCE = Positive Childhood Experience; Y = Youth Version Questionnaire | | | |

| Table S3. Measures of covariates. | | |
| --- | --- | --- |
| Covariates | TYP W1 Question/answer | Coding (#step) |
| Age | Q: [Y] birth year  Ans. _______ | #1. Calculation    2000 – birth year + 6/9 |
| Gender | Q: [Y] gender  Ans. 1.male 2.female | #1. Recode  0=male 1=female |
| Location | Q: [Y] residential area  Ans. 1.Taipei city 2.New Taipei city 3.I-Lan county | #1. Recode  0=Taipei city 1=New Taipei city 2=I-Lan county |
| Parental education | Q1: [P] my education attainment  Q2: [P] my spouse’s education attainment  Q3: [Y] my mom’s education attainment  Q4: [Y] my dad’s education attainment  Ans. 1.elementary school or below 2.junior high school 3.senior high school 4.vocational high school 5.junior college 6.university 7.graduate school or above | #1. Q1, Q2, Q3 & Q4 recode  0=below university 1=university or above  #2. Maximization (highest parental education)  P (Q1 & Q2); Y (Q3 & Q4)  #3. Mainly use P ans., compensated w/ Y ans. |
| Substance use | Q1: [Y] how often do you smoke or drink alcohol?  Q2: [Y] how often do you take drugs (e.g., superglue, amphetamine, etc)?  Ans. 1.never 2.sometimes 3.half of the time 4.mostly 5.always | #1. Q1 & Q2 recode  0=never 1=ever (others)  #2. Maximization  0=never 1=at least one substance use |
| Self-esteem | [Y] How much do you agree with the following description of yourself?  Q1: I am optimistic about myself.  Q2: I am satisfied with myself.  Q3: sometimes I feel useless.  Q4: sometimes I feel that I don’t have any desirable qualities.  Ans. 1.strongly agree 2.agree 3.disagree 4.strongly disagree | #1. Q1 & Q2 reverse code  #2. Average scores for four items  * higher score, higher level of self-esteem |
| Depressive symptoms | [Y] Did you have the following symptoms last week? If yes, how severe it is?  Q1: headache. Q2: dizziness. Q3: muscle pain. Q4: numbness/tingling pain in certain parts of the body. Q5: feeling like your throat is clogged. Q6: weakness in certain parts of the body. Q7: losing the will to live. Q8: loneliness. Q9: depressed. Q10: excessive worried.  Ans. 1.never 2.mild 3.moderate 4.serious 5.very serious | #1. Average scores for ten items  * higher scores, higher level of depressive symptoms |
| Insomnia symptom | Q: [Y] Did you sleep unwell or have insomnia symptom?  Ans. 1.never 2.mild 3.moderate 4.serious 5.very serious | #1. Recode  0=never 1=ever (others) |
| * TYP = Taiwan Youth Project; Y = Youth version questionnaire; P = Parental version questionnaire | | |

| Table S4. The results of the sample, with non-response items addressed through mean replacement (n = 2,903). | | | | | | | | | |
| --- | --- | --- | --- | --- | --- | --- | --- | --- | --- |
| Variables | Compensatory model |  | Protective model:  Stratification of PCE scores by mean split | | |  | Challenge model:  Stratification of ACE scores by four or more | | |
|  |  |  | PCE ≤ 3  n = 1,787 |  | PCE > 3  n = 1,116 |  | ACE < 4  n = 2,692 |  | ACE ≥ 4  n = 211 |
|  | Persistent insomnia  during emerging  adulthood (vs. N) |  | Persistent insomnia  during emerging  adulthood (vs. N) |  | Persistent insomnia  during emerging  adulthood (vs. N) |  | Persistent insomnia  during emerging  adulthood (vs. N) |  | Persistent insomnia  during emerging  adulthood (vs. N) |
|  | AOR [95% CI] |  | AOR [95% CI] |  | AOR [95% CI] |  | AOR [95% CI] |  | AOR [95% CI] |
| Age | 1.07 [0.95, 1.22] |  | 1.06 [0.91, 1.23] |  | 1.11 [0.90, 1.36] |  | 1.08 [0.95, 1.23] |  | 1.02 [0.67, 1.57] |
| Female (vs. male) | 1.28 [1.08, 1.51]** |  | 1.27 [1.03, 1.57]* |  | 1.24 [0.94, 1.65] |  | 1.24 [1.04, 1.48]* |  | 1.60 [0.88, 2.89] |
| **Early adolescence ^c^** |  |  |  |  |  |  |  |  |  |
| Location (vs. Taipei city) |  |  |  |  |  |  |  |  |  |
| New Taipei city | 1.03 [0.84, 1.25] |  | 1.08 [0.84, 1.39] |  | 0.94 [0.68, 1.29] |  | 1.04 [0.85, 1.28] |  | 0.89 [0.46. 1.73] |
| I-Lan County | 0.87 [0.70, 1.09] |  | 0.98 [0.74, 1.29] |  | 0.71 [0.48, 1.05]+ |  | 0.87 [0.69, 1.10] |  | 0.78 [0.34, 1.76] |
| Parental education (vs. below college) | 0.74 [0.57, 0.96]* |  | 0.69 [0.49, 0.99]* |  | 0.75 [0.51, 1.11] |  | 0.70 [0.53, 0.91]** |  | 1.31 [0.49, 3.50] |
| Substance use (vs. never) | 0.75 [0.55, 1.02]+ |  | 0.81 [0.57, 1.14] |  | 0.63 [0.32, 1.24] |  | 0.73 [0.52, 1.02]+ |  | 1.03 [0.48, 2.21] |
| Self-esteem | 1.02 [0.87, 1.21] |  | 1.10 [0.89, 1.36] |  | 0.86 [0.67, 1.11] |  | 1.03 [0.86, 1.23] |  | 0.95 [0.59, 1.54] |
| Depressive symptoms | 1.44 [1.23, 1.69]** |  | 1.42 [1.16, 1.73]** |  | 1.46 [1.13, 1.89]** |  | 1.58 [1.33, 1.88]** |  | 1.07 [0.74, 1.55] |
| Insomnia symptom | 1.75 [1.47, 2.09]** |  | 1.60 [1.28, 1.99]** |  | 2.07 [1.54, 2.78]** |  | 1.72 [1.43, 2.08]** |  | 1.75 [0.96, 3.19]+ |
| ACE | 1.11 [1.04, 1.18]** |  | 1.14 [1.06, 1.23]** |  | 1.07 [0.96, 1.20] |  | – |  | – |
| PCE | 0.93 [0.88, 0.99]* |  | – |  | – |  | 0.93 [0.88, 0.99]* |  | 0.82 [0.66, 1.02]+ |
| Note: p <0.1 (+); p <0.05 (*); p <0.01 (**)  AOR = Adjusted Odds Ratio; ACE = Adverse Childhood Experience; PCE = Positive Childhood Experience  ^a^ Challenge model of resilience theory: higher ACE diminished the protective effects of PCE on later persistent insomnia.  ^b^ Persistent insomnia was measured when subjects were around 20 and 22 years old.  ^c^ Early adolescence in this study was defined as the period when the subjects were around 14 years old. | | | | | | | | | |

| Table S5. The protective model of resiliency theory ^a^: Different cutoffs of PCE scores and their impact on the relationship between ACE and persistent insomnia during emerging adulthood ^b^ (n = 2,841). | | | | | | | | | | | |
| --- | --- | --- | --- | --- | --- | --- | --- | --- | --- | --- | --- |
| Variables | Stratification of PCE scores by 1 SD | | |  | Stratification of PCE scores by top 20% | | |  | Stratification of PCE scores by the highest two groups | | |
|  | PCE < 4  n = 1,754 |  | PCE ≥ 4  n = 1,087 |  | PCE < 5  n = 2,283 |  | PCE ≥ 5  n = 558 |  | PCE < 6  n = 2,620 |  | PCE ≥ 6  n = 221 |
|  | Persistent insomnia  during emerging  adulthood (vs. N) |  | Persistent insomnia  during emerging  adulthood (vs. N) |  | Persistent insomnia  during emerging  adulthood (vs. N) |  | Persistent insomnia  during emerging  adulthood (vs. N) |  | Persistent insomnia  during emerging  adulthood (vs. N) |  | Persistent insomnia  during emerging  adulthood (vs. N) |
|  | AOR [95% CI] |  | AOR [95% CI] |  | AOR [95% CI] |  | AOR [95% CI] |  | AOR [95% CI] |  | AOR [95% CI] |
| Age | 1.05 [0.90, 1.22] |  | 1.10 [0.89, 1.36] |  | 1.08 [0.95, 1.24] |  | 1.00 [0.73, 1.36] |  | 1.04 [0.91, 1.18] |  | 1.43 [0.84, 2.43] |
| Female (vs. male) | 1.28 [1.03, 1.58]* |  | 1.20 [0.90, 1.60] |  | 1.21 [1.00, 1.46]* |  | 1.38 [0.92, 2.07] |  | 1.21 [1.02, 1.45]* |  | 1.53 [0.80, 2.92] |
| **Early adolescence ^c^** |  |  |  |  |  |  |  |  |  |  |  |
| Location (vs. Taipei city) |  |  |  |  |  |  |  |  |  |  |  |
| New Taipei city | 1.11 [0.86, 1.42] |  | 0.96 [0.69, 1.32] |  | 1.07 [0.86, 1.34] |  | 1.00 [0.62, 1.60] |  | 1.05 [0.86, 1.29] |  | 0.98 [0.48, 1.99] |
| I-Lan County | 1.00 [0.76, 1.32] |  | 0.73 [0.49, 1.09] |  | 0.93 [0.72, 1.19] |  | 0.84 [0.47, 1.49] |  | 0.92 [0.72, 1.16] |  | 0.70 [0.28, 1.76] |
| Parental education (vs. < college) | 0.69 [0.49, 0.98]* |  | 0.75 [0.50, 1.11] |  | 0.77 [0.57, 1.03]+ |  | 0.55 [0.32, 0.97]* |  | 0.75 [0.57, 0.98]* |  | 0.38 [0.13, 1.06]+ |
| Substance use (vs. never) | 0.81 [0.57, 1.15] |  | 0.60 [0.30, 1.21] |  | 0.81 [0.59, 1.12] |  | 0.39 [0.10, 1.61] |  | 0.77 [0.56, 1.06] |  | 0.90 [0.15, 5.60] |
| Self-esteem | 1.10 [0.89, 1.36] |  | 0.89 [0.69, 1.15] |  | 1.11 [0.92, 1.33] |  | 0.69 [0.48, 0.99]* |  | 1.00 [0.84, 1.18] |  | 0.74 [0.44, 1.24] |
| Depressive symptoms | 1.42 [1.16, 1.74]** |  | 1.47 [1.14, 1.91]** |  | 1.51 [1.26, 1.81]** |  | 1.23 [0.87, 1.74] |  | 1.43 [1.21, 1.68]** |  | 1.71 [0.81, 3.59] |
| Insomnia symptom | 1.59 [1.27, 1.99]** |  | 2.13 [1.58, 2.87]** |  | 1.59 [1.31, 1.94]** |  | 2.79 [1.84, 4.23]** |  | 1.77 [1.47, 2.13]** |  | 1.89 [0.88, 4.03] |
| ACE | 1.15 [1.06, 1.24]** |  | 1.07 [0.96, 1.20] |  | 1.13 [1.06, 1.21]** |  | 1.10 [0.93, 1.30] |  | 1.11 [1.04, 1.19]** |  | 1.39 [0.94, 2.06] |
| Note: p <0.1 (+); p <0.05 (*); p <0.01 (**)  AOR = Adjusted Odds Ratio; ACE = Adverse Childhood Experience; PCE = Positive Childhood Experience  ^a^ Protective model of resilience theory: higher PCE mitigated the negative effects of ACE on later persistent insomnia.  ^b^ Persistent insomnia was measured when subjects were around 20 and 22 years old.  ^c^ Early adolescence in this study was defined as the period when the subjects were around 14 years old. | | | | | | | | | | | |

| Table S6. Different types of ACE on persistent insomnia during emerging adulthood ^a^. | |
| --- | --- |
| Variables | Persistent insomnia during emerging adulthood (vs. no) |
|  | AOR [95% CI] |
| **Early adolescence ^b^** |  |
|  |  |
| **Each Category** |  |
| Maltreatment ^c^ (vs. N) | 0.98 [0.82, 1.17] |
| Family dysfunction ^d^ (vs. N) | 1.31 [1.09, 1.57]** |
| Finkelhor et al. item ^e^ (vs. N) | 1.25 [1.04, 1.49]* |
|  |  |
| **Each type** |  |
| Emotional abuse (vs. N) | 1.26 [1.00, 1.58]* |
| Physical abuse (vs. N) | 1.24 [0.96, 1.60] |
| Emotional neglect (vs. N) | 0.90 [0.74, 1.11] |
| Parental divorce/separation (vs. N) | 1.49 [1.10, 2.03]* |
| Household substance abuse (vs. N) | 1.20 [0.93, 1.54] |
| Household mental illness (vs. N) | 1.41 [1.10, 1.80]** |
| Incarcerated household member (vs. N) | 0.85 [0.45, 1.63] |
| Peer isolation/rejection (vs. N) | 1.23 [1.00, 1.51]+ |
| Community violence (vs. N) | 1.47 [1.12, 1.92]** |
| Low socioeconomic status (vs. N) | 1.04 [0.81, 1.33] |
| Note: p <0.1 (+); p <0.05 (*); p <0.01 (**)  AOR = Adjusted Odds Ratio; ACE = Adverse Childhood Experience; PCE = Positive Childhood Experience  The models of persistent insomnia during emerging adulthood with different types of ACE were all adjusted for age, gender, location, parental education, substance use, self-esteem, depressive symptoms, prior insomnia symptoms, and accumulation of PCE.  ^a^ Persistent insomnia was measured when subjects were around 20 and 22 years old.  ^b^ Early adolescence in this study was defined as the period when the subjects were around 14 years old.  ^c^ Maltreatment: ever experienced emotional abuse, physical abuse, and emotional neglect.  ^d^ Family dysfunction: ever experienced parent divorce/separation, substance abuse, mental illness, and incarcerated members in the household.  ^e^ Finkelhor et al. item: ever experienced peer isolation/rejection, community violence, and low SES. | |

| Table S7. Different types of PCE on persistent insomnia during emerging adulthood ^a^. | |
| --- | --- |
| Variables | Persistent insomnia during emerging adulthood (vs. no) |
|  | AOR [95% CI] |
| **Early adolescence ^b^** |  |
| Able to talk to family about feelings (vs. N) | 0.92 [0.76, 1.13] |
| Felt family stood by them during difficult times (vs. N) | 0.96 [0.79, 1.15] |
| Felt safe and protected by adult in difficult times (vs. N) | 1.01 [0.83, 1.22] |
| Had at least 2 non-parent adults who took genuine interest them (vs. N) | 0.80 [0.66, 0.97]* |
| Felt supported by friends (vs. N) | 0.77 [0.65, 0.91]** |
| Felt a sense of belonging at high school (vs. N) | 0.82 [0.69, 0.98]* |
| Enjoyed participating in community traditions (vs. N) | 1.00 [0.84, 1.19] |
| Note: p <0.1 (+); p <0.05 (*); p <0.01 (**)  AOR = Adjusted Odds Ratio; ACE = Adverse Childhood Experience; PCE = Positive Childhood Experience  The models of persistent insomnia during emerging adulthood with different types of PCE were all adjusted for age, gender, location, parental education, substance use, self-esteem, depressive symptoms, prior insomnia symptoms, and ACE.  ^a^ Persistent insomnia was measured when subjects were around 20 and 22 years old.  ^b^ Early adolescence in this study was defined as the period when the subjects were around 14 years old. | |
